# Review on the Applications of Biomass-Derived Carbon Materials in Vanadium Redox Flow Batteries

**DOI:** 10.1021/acsomega.3c03648

**Published:** 2023-09-14

**Authors:** Hilal Doǧan, Mert Taş, Tuǧba Meşeli, Gülşah Elden, Gamze GENC

**Affiliations:** † Department of Energy Systems Engineering, Faculty of Engineering, Erciyes University, Kayseri 38039, Turkey; ‡ Energy Systems Engineering Program, Graduate School of Natural and Applied Sciences, Erciyes University, Kayseri 38039, Turkey; § Energy Conversions Research and Application Center, Erciyes University, Kayseri 38039, Turkey; ∥ Electrochemical Storage and Energy Conversion Laboratory, Erciyes University, Kayseri 38039, Turkey

## Abstract

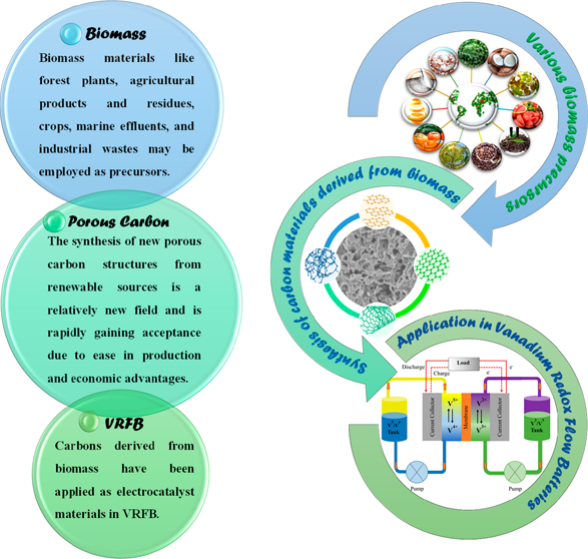

The development of vanadium redox flow batteries (VRFBs) requires the exploration of effective and affordable electrodes. In order to increase the electrochemical activity of these electrodes and decrease the polarizations, they are doped with an electrocatalyst. In this context, the use of biomass-derived materials as electrocatalysts in VRFBs has received much attention recently due to their widespread availability, renewable nature, low cost, and high energy efficiency. This paper aims to review the synthesis methods of biomass-derived carbon materials and their applications in VRFBs. In line with this aim, recent developments in carbon-based electrode modification methods and their electrochemical performance in VRFBs are summarized. The studies show that porous carbon electrocatalysts increase energy efficiency by reducing overpotentials and improving electrocatalytic activation. In addition, it is thought that biomass carbon doped electrocatalysts can improve the hydrophilicity of the electrodes, the transfer of vanadium ions, and the reaction kinetics. The highest charge voltage decrease rate of 8.61% was obtained in the *Scaphium scaphigerum*, whereas the highest discharge voltage increase rate of 14.29% was observed in the twin cocoon, as in all reviewed studies. Furthermore, the maximum energy efficiency (75%) was achieved in a VRFB equipped with an electrode doped with carbon derived from *Scaphium scaphigerum* and cuttlefish. It can be concluded from the reviewed studies that the electrochemical performances of electrodes doped with biomass-derived carbons in VRFBs are more effective than those of the bare electrodes.

## 1 Introduction

Most of the world’s energy needs are met from fossil fuels at a rate of 86%, and this causes a lot of important problems such as global warming, depletion of the ozone layer, pollution, acid rain, etc. For this reason, researchers have focused on investigating clean and alternative renewable energy sources. Taking into consideration environmental, economic, social, and energetic factors, renewable energy is the most promising solution to deal with the issues mentioned above in future processes for energy production and storage. However, some renewable energy sources such as wind and solar energy having high potentials are not continuous. To overcome this issue, energy storage devices are integrated into the system, and so the use rate of renewable energy is increased. While the current energy storage capacity is less than 3% nowadays, this rate can be increased by using safe, sustainable, efficient, and large-scale energy storage systems.

Energy storage systems can be classified as mechanical, thermal, electrochemical, electrical, and chemical.^1,2^ Recent studies have been focused on electrochemical energy storage systems that have increased in number and size. Among these electrochemical storage systems, especially redox flow batteries (RFBs) have attracted the most attention because of their long duration, scalability, and nonflammability.^3−5^ RFBs can be made using a variety of redox couples including vanadium-vanadium, vanadium-bromine, vanadium-oxygen, vanadium-cerium, vanadium-polyhalite, bromine-polysulfide, zinc-bromine, zinc-cerium, zinc-iron, iron-chromium, magnesium-vanadium, and hydrogen-bromine.^6−8^ Among these, the all-vanadium chemistry used in a vanadium redox flow battery (VRFB) is by far the most advanced option due to its good properties such as high energy efficiency, high power density, wide operating temperature range, low capital cost, low toxicity, and long life cycle.^9−11^ A VRFB consists of a current collector, electrode, electrolytes (VO^2+^/VO_2_^+^ and V^2+^/V^3+^ sulfate solution for positive and negative electrolytes, respectively), membrane, gasket, electrolytic tank, and peristaltic pump.^12−14^ During the charge and discharge processes, vanadium species undergo chemical reactions via reversible redox reactions. Since these reactions occur at the electrode–electrolyte interface, the energy efficiency of a VRFB mainly depends on the electrodes. The electrical conductivity of the electrode affects the ohmic polarization of a VRFB because electron and vanadium ion transfer occurs on electrode surfaces. Also, the mechanical and chemical stability of the electrode has a significant impact on the battery’s life and performance.^15,16^ The most used electrode materials in VRFBs are carbon-based materials, such as graphite felt, carbon felt, and carbon paper. In particular, carbon and graphite felt materials have properties of low-cost availability, a wide operating range, good chemical stability, high surface area, and porosity. However, these electrode materials have limited the energy efficiency in VRFBs because of their low electrochemical activity and poor electrode wettability.^17^ Therefore, current research on high-performance VRFBs is focused on various electrode modification approaches to boost the properties of electrodes, such as specific surface area, wetting capacity, electrical conductivity, and reaction kinetics. These modifications mainly include various physical and chemical activation methods, including heat treatment, acid treatment, and electrocatalyst doping,^18−20^ to improve the electrochemical activity of the electrodes. The heat treatment and acid treatment can enhance the electrode’s chemical activity due to the fact that the oxygen functional groups (−OH, −COOH, and CO) produced on the electrode after such treatments become active sites, which can boost the electrode’s hydrophilicity and bump up the accessibility of the electrolyte.^18,21^ Another treatment is to dope metal compounds or carbon nanomaterial electrocatalysts onto the electrode surface. There have been studies on the use of various metal or metal oxide based (Bi,^22−24^ Fe,^25^ Cu,^26^ Pt,^27^ Ir,^28^ TiO_2_,^29^ WO_3_,^30,31^ CoO,^32^ Cr_2_O_3_,^33^ SnO_2_,^34^ NiO,^35^ etc.) electrocatalysts to modify carbon and graphite felt electrodes.

The use of metal oxides as catalysts provides two important contributions for vanadium redox reactions. First, these catalysts increase active sites along the surface of the electrode, which facilitate the adsorption and reaction of vanadium species. In addition, these active sites can promote the transfer of electrons in the vanadium redox system. Second, the hydrophilic nature of metal–oxygen bonds in metal oxide catalysts plays a crucial role in promoting the mass transfer of reactants and products. The hydrophilic metal–oxygen binding can improve the solubility of vanadium species, allowing for easier diffusion and transport of ions within the catalytic system. This improvement in mass transfer enhances the overall efficiency of the vanadium redox reactions. To further enhance the catalytic performance of metal oxide catalysts, partial reduction and doping techniques can be used to modify the catalytic properties.^36−38^ Even though noble metals increase electrical conductivity and exhibit excellent performance, their high cost hinders their applications. The transition metal oxide catalysts increase the number of active sites, and most of them are relatively unstable in acidic solutions.^39^ Therefore, researchers have focused on an approach which is the implementation of nonmetallic heteroatoms such as nitrogen, boron, phosphorus, and sulfur.^40^ This approach has shown a significant improvement in the electrochemical performance of carbon materials as a result of improved ionic diffusion and electron transport and increased numbers of oxygen functional groups. Carbon nanotubes,^41^ carbon nanofibers,^42^ graphene nanosheets,^43^ and graphene oxides^44^ have all been studied as electrocatalysts. While carbon-based electrocatalysts increase the electrode’s specific surface area, they cause the low wettability of the electrode and consequently inferior cycle stability.^45,46^

Many studies have been conducted on VRFBs using various methods to improve the performance of electrode materials, and most of these studies were focused on different modification approaches based on nonrenewable precursors.^47−49^ However, nowadays, studies have shifted toward renewable precursors. Biomass and biomass waste carbons, which are new types of environmentally friendly carbon materials, are emerging as potential precursors for the development of high-performance, green, renewable carbon materials.^50^ Biomass-derived carbon materials (B-CMs) have attracted much attention over conventional carbon materials among energy storage materials because they have many advantages as follows: (i) the raw materials are natural and do not pollute the environment, (ii) the biomass conversion method is simple, and (iii) the structure is diversified to meet various needs in different areas.

The focus of this review is to provide the recent research on the B-CMs used in VRFBs. In the first stage, characterizations of B-CMs are discussed according to the synthesis procedure properties, such as biomass precursors, activation agent, inert gas, temperature, and residence time. In another stage, the application of B-CMs as electrocatalyst in VRFBs and their effect on the performance are presented. The main novelty of the paper is the extensive evaluation of B-CMs used as electrocatalysts in VRFBs, and exhibiting especially how the potential performance, charge–discharge capacities, and all efficiencies are affected in VRFBs.

## 2 Research Trends in Biomass Energy: A Bibliometric Analysis

**Figure 1:**
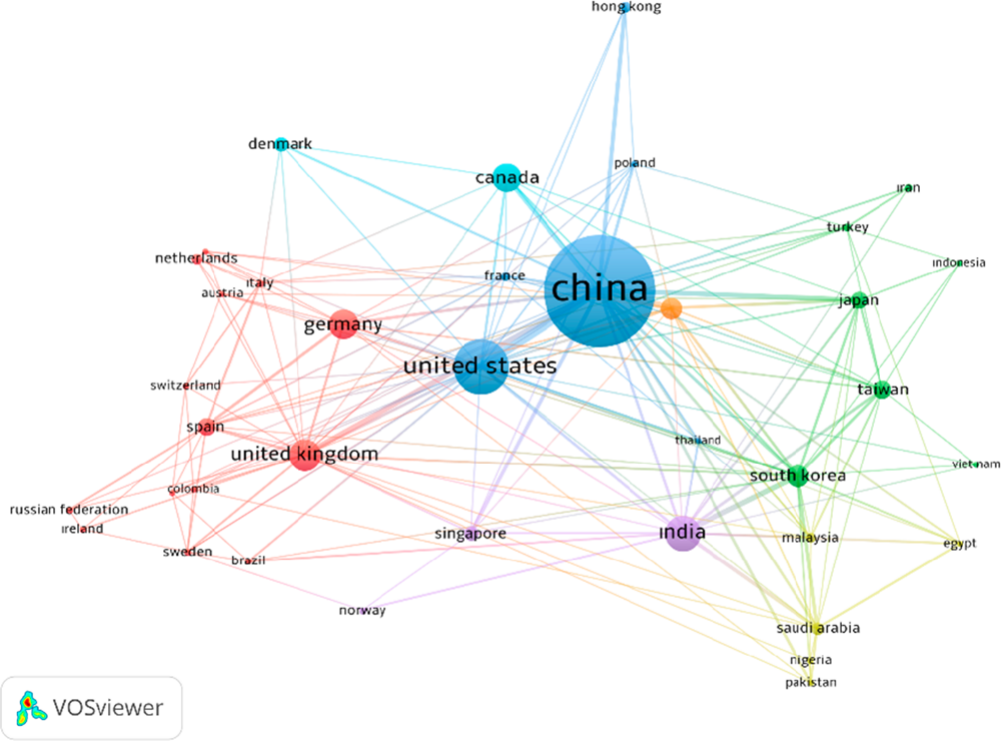
Network visualization map of the distribution of biomass carbon studies for the period in recent years by country.

In this presented study, first, a simple bibliometric analysis based on the data obtained from the Scopus database was carried out to reveal improvements and interest over years in biomass-derived carbon materials. For this analysis, the papers were searched with the keywords “Biomass Carbon” and “Energy Storage” in the Scopus database. The VOS viewer program was used to show the distribution of the articles on biomass carbon in energy storage according to the countries in which they were carried out (according to the number of publications and citations) and to show the relations between countries. Figure 1 shows the countries that contributed to the articles in biomass-derived carbon studies. As can be seen in Figure 1, China is the most productive country with the highest number of publications in the field of study on biomass carbon. The publications presented by China are in existing collaboration with each member of the network. The United States, United Kingdom, and India come next, respectively, in terms of density following China.

**Figure 2:**
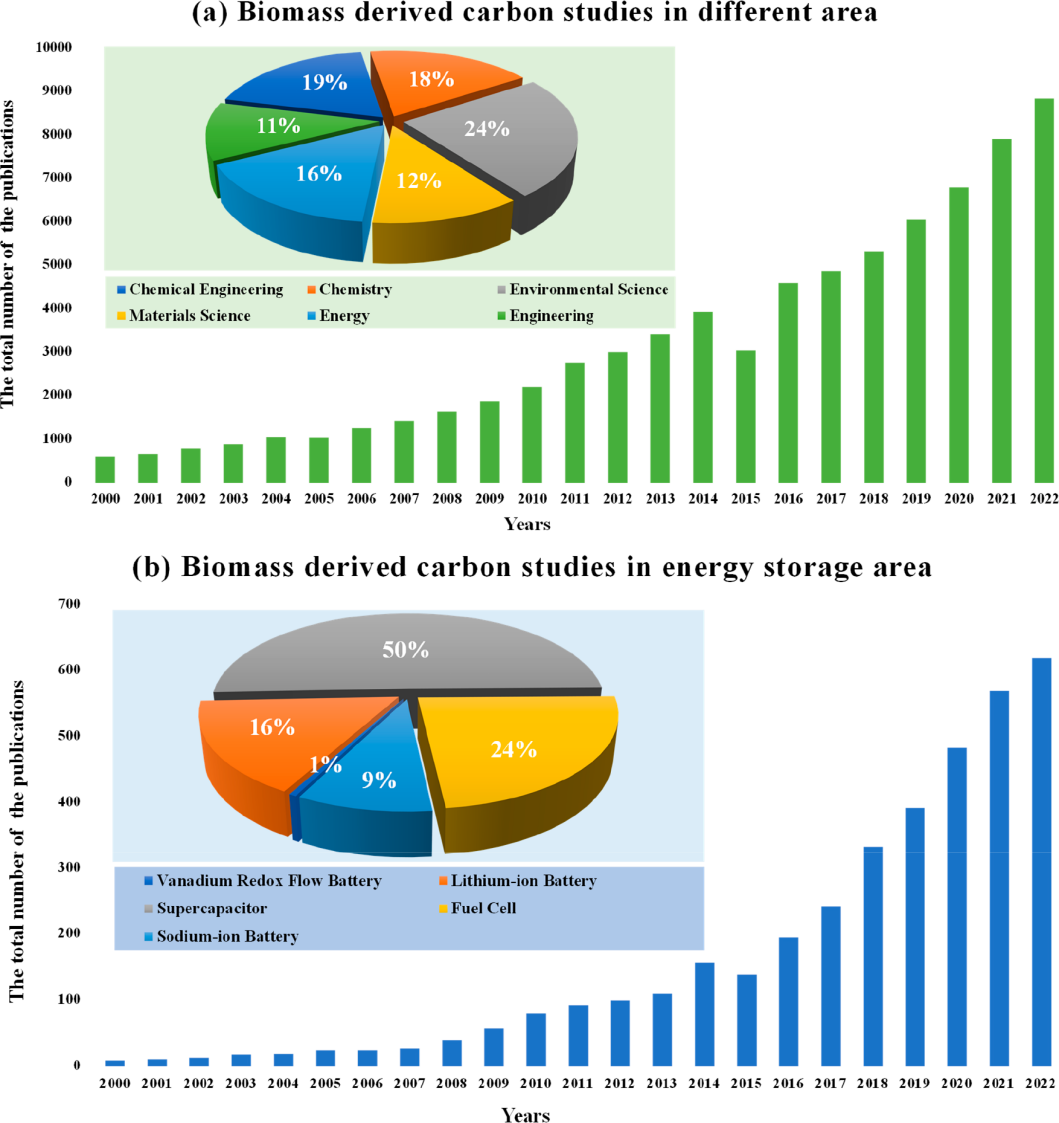
Total number of the publications and the proportions according to the research areas since 2000 for (a) biomass-derived carbon in general and (b) biomass-derived carbon in energy storage.

Second, in order to reveal the biomass-derived carbon electrode material development process, the indexed papers in the last 12 years were searched with the keywords “Biomass Carbon and Energy Storage” and the data found are plotted in Figure 2. While Figure 2a depicts the trends in biomass-derived carbon studies versus years as well as the share distribution of biomass carbon studies in the considered areas (Chemical Engineering, Chemistry, Environmental Science, Materials Science, Energy and Engineering), Figure 2b demonstrates the number of papers related to B-CM applications in various energy storage systems and their share distribution. It is clearly observed from both subfigures that the application of biomass-derived carbon in areas of both the general uses and energy storage is continuing to grow unabated. According to Figure 2a, B-CMs especially began to be studied extensively after 2007. The applications of sustainable carbon materials have received increasing attention, particularly in the context of the energy/chemical industry in addition to traditional environmental science. As is apparent from Figure 2b, supercapacitors are noted to be the first research area with many publications in B-CMs and its proportion is 50% of the overall studies. This is followed by fuel cells and lithium-ion batteries with values of 24%, and 16%, respectively. While the proportion of the papers related to B-CMs in VRFBs over the studies carried out to date is only 1%, it is expected that this proportion will increase gradually. This is because, lately, B-CMs have gained more and more attention as electrode materials in VRFBs due to their cost-effectiveness and environmental friendliness as well as their high adsorption, fast ion/electron transport, and unique tunable physicochemical properties.^51−69^

## 3 Synthesis Methods of Biomass-Derived Carbon Materials

**Figure 3:**
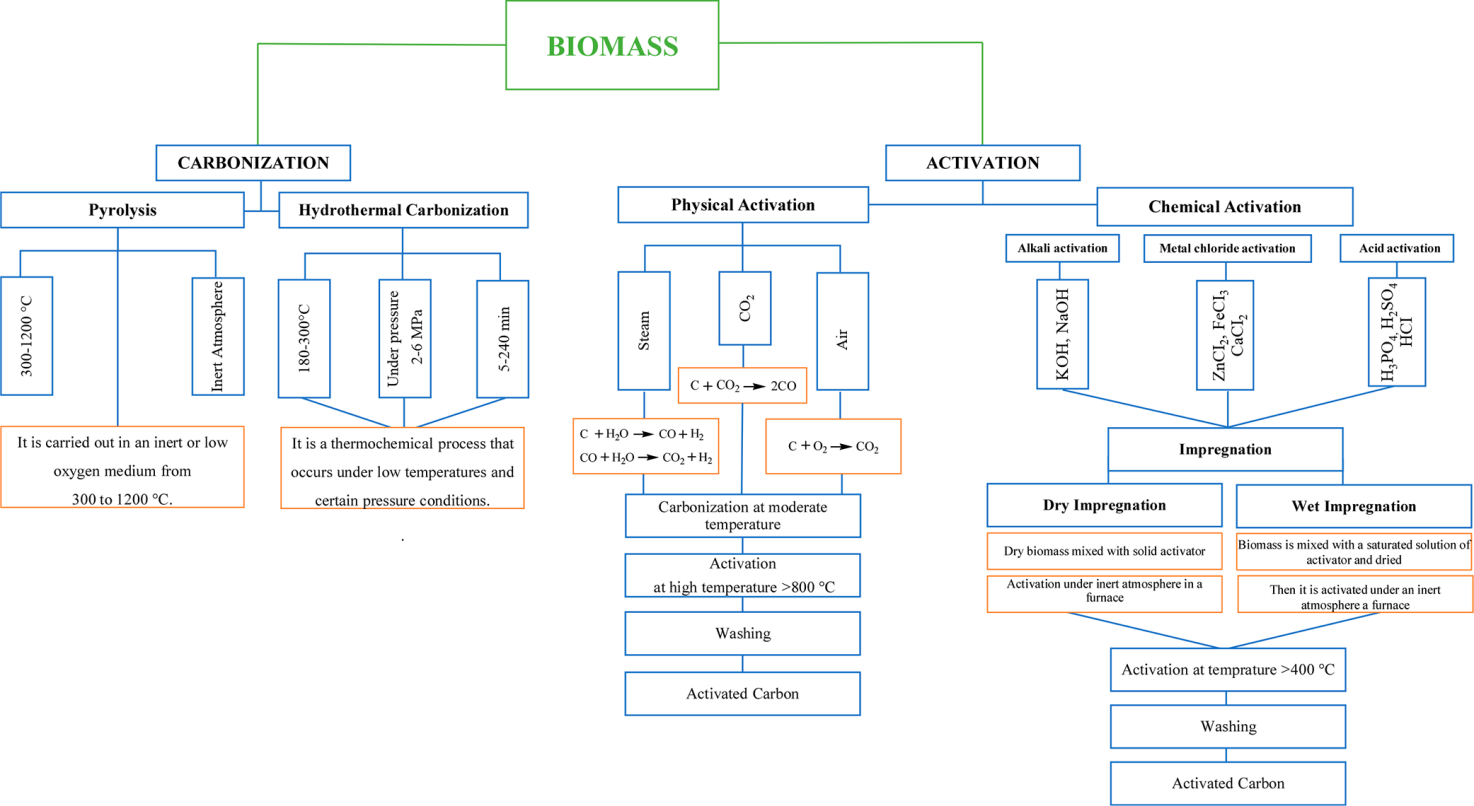
Illustration of the synthesis methods of biomass-derived carbon materials.

B-CMs, which consist of interconnected hierarchical, multidimensional, and porous structures, have many outstanding properties such as high performance, environmental friendliness, abundance, and renewability. Although B-CMs are commonly employed as electrocatalysts in energy storage systems because of these features, there are only a few studies related to B-CM applications in VRBF systems. The attraction of B-CMs exhibiting considerable electrochemical features increases day by day in VRFB applications. The studied biomasses to produce B-CMs and their synthesis methods are given in Table 1 in detail. As is apparent from this table, B-CMs are mainly synthesized by carbonization and activation procedures. Ar_2_ and N_2_ are usually preferred as the inert gas, while activation agents are KOH, ZnCl_2_, FAC, K_2_FeO_4_, (NH_4_)_2_C_2_O_4_, H_3_PO_4_, and Na_2_CO_3_ in these procedures. Also, the operating temperature changes from 80 to 1000 °C according to the biomass type and the selected synthesis method. The procedures of carbonization and activation methods are illustrated in Figure 3. It is seen from this figure that the carbonization method is classified as hydrothermal carbonization and pyrolysis and the activation method is classified as physical and chemical activations.

**Table 1:** Synthesis Methods of Biomass-Derived Carbon Materials Studied in VRFBs

biomass	method	activation agent	ambient temperature	inert gas	ref
kiwifruit	hydrothermal carbonization	ferric ammonium citrate	180 °C/12 h	Ar_2_	(51)
	pyrolysis		800 °C/2 h		
*Scaphium scaphigerum*	hydrothermal carbonization	K_2_FeO_4_	180 °C/15 h	Ar_2_	(52)
	pyrolysis		800 °C/2 h		
persimmon	hydrothermal carbonization	(NH_4_)_2_C_2_O_4_	140 °C/10 h	Ar_2_	(53)
	pyrolysis		750 °C/3 h		
shaddock peel	hydrothermal carbonization	H_3_PO_4_	180 °C/12 h	Ar_2_	(54)
	pyrolysis		800 °C/3 h		
twin-cocoon	hydrothermal carbonization	Na_2_CO_3_	120 °C/2 h	Ar_2_	(55)
	pyrolysis		1000 °C/4 h		
shrimp-shell-extracted chitin/pine wood	hydrothermal carbonization		200/6 h	N_2_	(56)
	pyrolysis		850 °C/2 h		
fungi	hydrothermal carbonization	KOH	180 °C/24 h	Ar_2_	(57)
	pyrolysis		800 °C/2 h		
fish scales	hydrothermal carbonization	KOH	80 °C/24 h	Ar_2_	(58)
	carbonization		700 °C/2 h		
*Scaphium scaphigerum*	hydrothermal carbonization	FeCl_3_	180 °C/15 h	Ar_2_	(59)
	pyrolysis		800 °C/2 h		
*Scaphium scaphigerum*	hydrothermal carbonization	urea	180 °C/36h	Ar_2_	(60)
	pyrolysis		850 °C/2 h		
sugar cane bagasse	precarbonization	KOH	230 °C/12 h	N_2_	(61)
	pyrolysis		700 °C/2 h		
coconut shells	hydrothermal carbonization	ZnCl_2_	275 °C/20 min	N_2_/CO_2_	(62)
	physical activation		800 °C/2 h		
coffee beans	pyrolysis		850 °C/30 min	N_2_	(63)
	physical activation		850 °C/3 h	N_2_/steam	
cuttlefish bone	pyrolysis		600 °C/1 h	Ar_2_	(64)
	physical activation		300 °C/1,3,8 h	air	
black tea bags	pyrolysis	KOH	600 °C/3 h	N_2_	(65)
	chemical activation		800 °C/1 h	N_2_	
orange peel	pyrolysis	KOH	400 °C/1 h	Ar_2_	(66)
	chemical activation		800 °C/2 h	Ar_2_	
sal wood sawdust	hydrothermal carbonization	ZnCl_2_	275 °C/20 min	N_2_	(67)
	physico-chemical activation		850 °C/2 h		

Carbonization is a heating process in which an organic material is converted into a carbon-rich coal-like material under oxygen-deficient or oxygen-free conditions. The solid product obtained from the carbonization of biomass is known as biochar. The quality of biochar is significantly influenced by biomass characteristics, such as biomass type and chemical composition, especially lignin content, particle size, moisture, and mineral salt contents. Furthermore, its quality is also dependent on carbonization conditions such as heating rate, residence time, and temperature.^70−72^ Although there are several carbonization procedures, pyrolysis and hydrothermal carbonization are the most widely employed to produce biochar in the literature. *Pyrolysis* is the thermal decomposition of biomass in the absence of oxygen at operating temperatures ranging from 400 to 1200 °C. It is known that a slow heating rate, low operating temperature, and long residence time during the pyrolysis process give the a higher yield of biochar.^70^*Hydrothermal carbonization (HTC)*, also called wet pyrolysis, takes place between 180 and 300 °C on immersion in water or aqueous solutions from 5 to 240 min under pressure (2–6 MPa). The main product acquired with HTC is called hydrochar, and it exhibits extremely hydrophobic and brittle properties as a result of including a high content of oxygen functional groups. The yield of hydrocar is significantly affected by temperature, heating rate, and residence time similarly to pyrolysis.^73−75^ Hydrochar/biochar derived from biomass via pyrolysis, carbonization, or both carbonization processes usually has a very low specific surface area, pore diameter, and pore volume. In order to improve these properties, a two-step process coordinating the activation process and carbonization can be useful.^76^ As mentioned above, the activation process can occur in two different ways, which are physical and chemical activation.

*Physical activation* is a process in which the porous structure of biochar is improved, and this process occurs between 350 and 1000 °C by using activating agents such as air, steam, O_2_, CO_2_, or their mixtures.^77,78^ In case of using steam as an activation agent, there is a high operating temperature requirement (above 750 °C) in the reaction taking place between steam and biochar. However, when this activation occurs at high temperatures of over 900 °C, excessive steam penetrating into carbon particles prevents the homogeneity of the reaction happening between the activation agent and biochar. This means that the reaction cannot proceed properly. Therefore, a low activation temperature must be preferred for the development of porosity and the increase in surface area. Since the activation rate of CO_2_ among the activation agents is relatively slower, the activation process with CO_2_ is easier in order to control the specific surface area and pore structure of carbon materials by adjusting the activation time. When O_2_ or air is preferred as the activation agent, many complications can occur in the exothermic reactions happening between carbon/O_2_ or carbon/air. Specifically, an increase in the reaction rate promotes excessive combustion and a lower activated carbon efficiency. Also, it is difficult to control the reaction mechanisms, the specific surface area, and the pore structure of the carbon materials. Because both CO_2_ and steam contribute to improvements of microporosity in the carbon material, they are increasingly being employed. The *chemical activation process* consists of the impregnation of chemical agents into the carbon precursor (such as potassium hydroxide (KOH), zinc chloride (ZnCl_2_), sodium hydroxide (NaOH), phosphoric acid (H_3_PO_4_), potassium chloride (KCl), and nitric acid (HNO_3_)) and heat treatment in the temperature range of 400–900 °C. The final product obtained from the chemical activation is washed to reveal porosity and to remove the impregnated activating agent. Chemical activation has many advantages over physical activation. These advantages are as follows: (i) reaction takes place at lower temperatures in one step, (ii) a higher carbon yield is obtained, (iii) materials with a high surface area are synthesized, (iv) the pore size development is good, and (v) the pore size is controllable. However, chemical activation can exhibit disadvantages such as an increase in the cost of the process because of activation agents and the time-consuming postactivation, in which the product is washed to remove impurities.^79^ Furthermore, the main parameters of chemical activation are the activation agent, impregnation ratio, activation temperature, and activation time. With respect to the reviewed studies, the recommended values of these parameters are as follows: the activation temperature is in the range of 550–900 °C, the impregnation ratio ranges from 1:2 to 1:5 (sample:activating agent), the heating rate is between 3 and 10 °C min^–1^, and the activation time changes from 1 to 4 h. It is shown that these parameters significantly affect the carbon yield, the formation of pores in the carbon, and the expansion of the surface area.^76,80,81^

## 4 Characterization of Biomass-Derived Carbon Materials

As mentioned above, the main purpose of porous carbon synthesis studies is to provide high surface area and porosity by composing optimum reaction conditions with appropriate raw materials and activation agents. Detailed information (such as biomass precursors, activation agent, inert gas, temperature, and residence time) can be obtained from Table 1 regarding synthesis methods of the B-CMs discussed in the literature.

**Figure 4:**
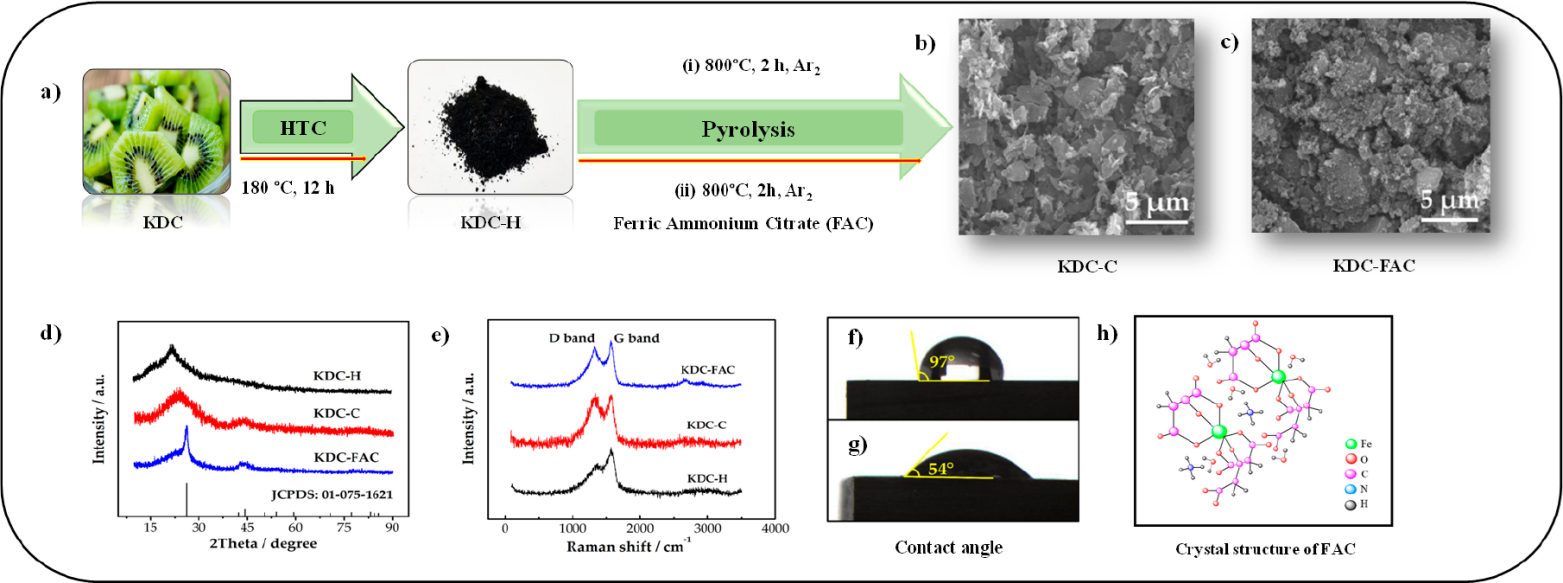
(a) Synthesis scheme of porous carbon from kiwi biomass. (b, c) SEM images of KDC-C and KDC-FAC. (d) XRD curves of KDC-H, KDC-C, and KDC-FAC. (e) Raman spectra of KDC-H, KDC-C, and KDC-FAC. (f, g) Contact angle presentation of KDC-C and KDC-FAC, respectively. (h) Crystal structure of FAC. Reprinted with permission from ref (51). Copyright 2020 Elsevier.

Cheng et al.^51^ synthesized extremely graphitized nitrogen (N)-doped porous carbon from kiwifruit as a biomass precursor. In order to produce this carbon material, after the hydrothermal carbonization along 12 h at 180 °C, high-temperature pyrolysis was realized at 800 °C in an Ar_2_ atmosphere as shown in Figure 4. Porous carbons produced from two pyrolysis cases were examined, i.e.: (i) the biomass-based carbon produced from direct pyrolysis without an activation agent (KDC-C) and (ii) using ferric ammonium citrate (FAC) activation agent in the pyrolysis process (KDC-FAC). When the SEM images of KDC-C and KDC-FAC samples are compared, the KDC-FAC structure has more homogeneous nanoparticles. It was seen from an XRD analysis of the two products that KDC-C had two broad peaks at 24 and 44° while KDH-FAC had a narrow peak at 26° and a broad peak at 44°. Moreover, the peak of KDC-FAC at 26° was narrower than those for KDC-C. Though the peak values in the D and G bands for both products were about 1350 and 1580 cm^–1^, respectively, the intensity ratios of the G band to the D band (*I*_G_/*I*_D_) used to characterize the crystallinity of carbon materials in KDC-C and KDC-FAC were 1 and 1.14, respectively, according to the Raman spectroscopy analysis. It can be understood from all results that KDC-FAC has a higher degree of graphitization than KDC-C. This is due to the fact that the FAC activation agent causes a greater graphitization on carbon compounds. FAC, which is used as an iron precursor, nitrogen supply, and reduction agent in hydrochar, offers iron species loading and nitrogen doping. During pyrolysis, iron reacting with carbon penetrates to the structure and it contributes to more porosity of the structure. Ammonium in FAC forms N-containing functional groups. Therefore, a high degree of graphitization occurs by forming a hierarchical porous structure. KDC-FAC having a contact angle of 54° provides better wettability than KDC-C having a contact angle of 97° (Figures 4f,g).

**Figure 5:**
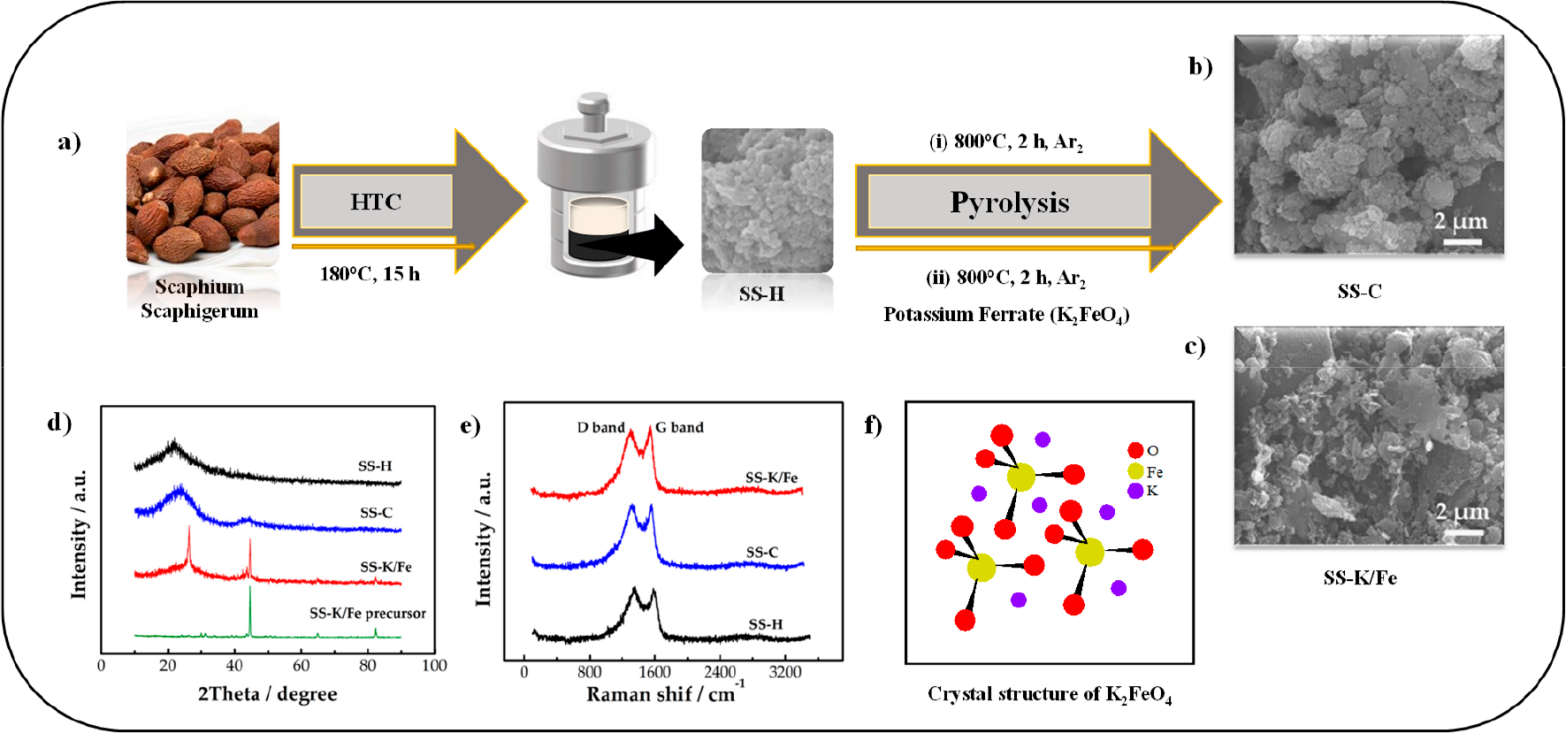
(a) Synthesis scheme of porous carbon obtained from *Scaphium scaphigerum*. (b, c) SEM images of SS-C and SS-K/Fe, respectively. (d) XRD curves of SS-H, SS-C, and SS-K/Fe. (e) Raman spectra of SS-H, SS-C, and SS-K/Fe. (f) Crystal structure of FAC. Reprinted with permission from ref (52). Copyright 2020 Elsevier.

Lv et al.^52^ used the *Scaphium scaphigerum* (SS) biomass precursor to produce the porous carbon. The synthesis scheme of porous carbon is given in Figure 5. As can be seen from this figure, first, the HTC method was carried out for 15 h at 180 °C. Then, the pyrolysis process was applied to the hydrochar (SS-H) at 800 °C with potassium ferrate (K_2_FeO_4_) activation agent and the final product (SS-K/Fe) was obtained. A set of surface analyses for porous carbons (SS-C and SS-K/Fe) were performed. It was seen from SEM analysis, while SS-K/Fe consisted of a mixture of nanosheets and nanoparticles, SS-C was only composed of nanoparticles. In addition, the peaks of SS-C and SS-K/Fe in XRD analysis were observed at 24 and 44° and at 26 and 44°, respectively. The low-intensity peak values in the D band for SS-C and SS-K/Fe were 1340 cm^–1^, whereas the peak values in the G band were 1600 cm^–1^. Besides, the *I*_D_/*I*_G_ values of SS-C and SS-K/Fe were 1.01 and 0.97, respectively. These results showed that the porous carbon (SS-K/Fe) having a higher graphitization degree and larger surface area was produced with use of the K_2_FeO_4_ agent. This is because K_2_FeO_4_ gradually decomposes into various solid and gaseous substances such as iron hydroxide (Fe(OH)_3_), potassium hydroxide (KOH), and oxygen (see eq 1)^82^ in an aqueous medium with increasing temperature. Thus, the revealed KOH provides the formation of oxygen-containing groups, resulting in a porous structure, and Fe(OH)_3_ contributes to the high graphitization of the carbon structure.
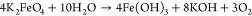
1

**Figure 6:**
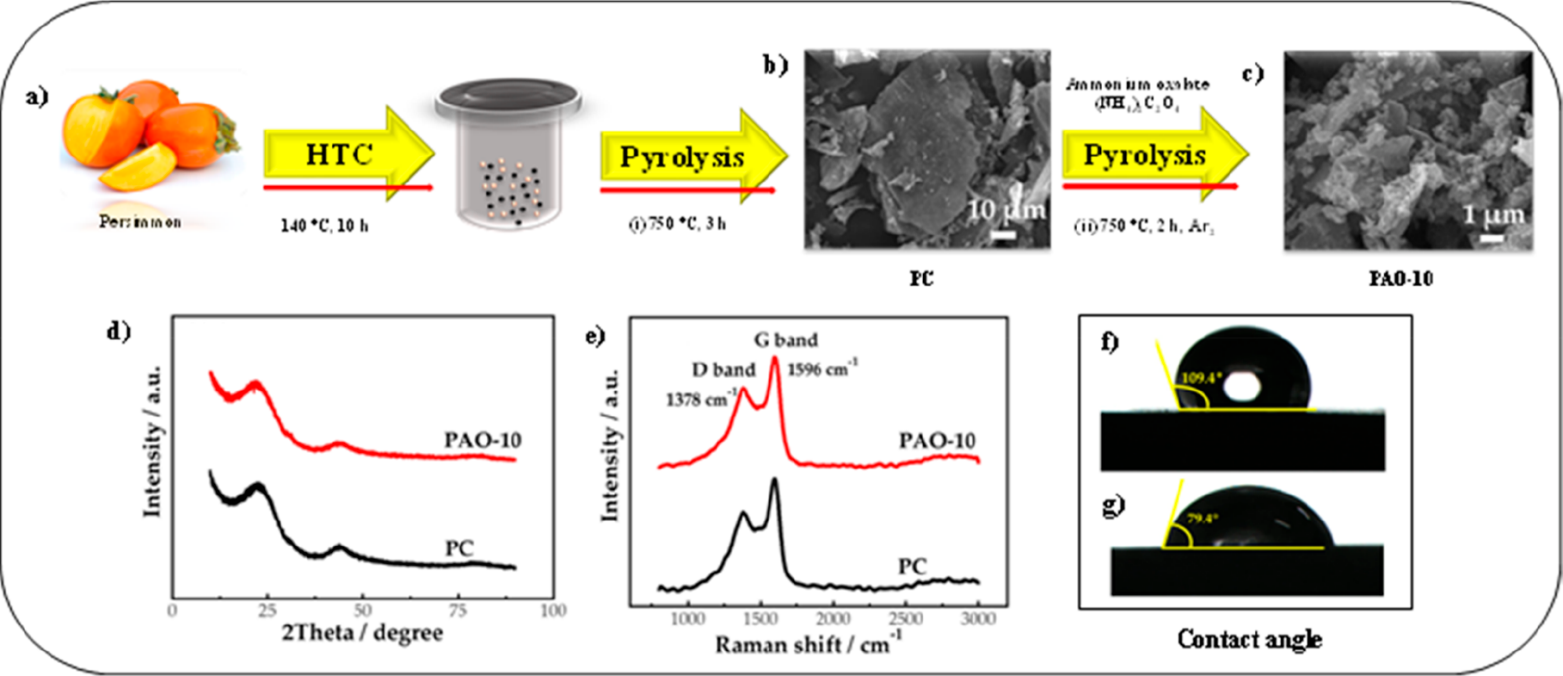
(a) Schematic illustration of persimmon-derived carbon material obtained by a hydrothermal and carbonization process. (b, c) SEM images of PC and PAO-10, respectively. (d) XRD curves of PC and PAO-10. (e) Raman spectra of PC and PAO-10. and (f, g) Contact angle presentation of PC and PAO-10, respectively. Reprinted with permission from ref (51). Copyright 2021 Springer Nature.

N-doped carbon materials synthesized from persimmon precursor were produced by Zhang et al.^53^ for use as a catalyst for VRFBs. Figure 6 illustrates the synthesis steps of porous carbon from persimmon. The hydrothermal carbon obtained by applying hydrothermal carbonization to the persimmon precursor was directly exposed to a pyrolysis process at 750 °C for 3 h under an argon atmosphere, and this product was named preliminary carbonization (PC). Just after this process, N-doped biomass carbon material was achieved by impregnating (NH_4_)_2_C_2_O_4_ activation agent to PC at different ratios (1:5, 1:10, 1:15) at 750 °C for 2 h. It was found from SEM analysis that the best carbon skeleton for PC (PAO-10) was for a ratio of 1:10 in comparison to PC. Another important result is that the *I*_D_/*I*_G_ ratios were computed as 0.72 and 0.70, respectively, for PAO-10 and PC, in accordance with the Raman analysis results. These results indicated that PAO-10 had a higher degree of defects than PC. Moreover, it was revealed from XRD analysis results that PAO-10 had the highest degree of defects. The contact angles of PC and PAO-10 are illustrated in Figures 6f,g. The contact angles of PC and PAO-10 are 109.4 and 79.4°, respectively. It can be understood that PAO-10 is hydrophilic while PC is hydrophobic.

Similarly, in other studies where synthesized porous carbon was obtained from shaddock peel,^54^ twin-cocoon,^55^ pinewood/chitin,^56^ fungi,^57^ fish scales,^58^*Scaphium scaphigerum*,^59,60^ and sugercane bagasse,^61^ these biomasses were subjected to a hydrothermal process first, followed by pyrolysis. It was observed clearly in these studies that two diffraction peaks appeared in the center ranges 2θ = 22.4–26.5° and 2θ = 43–44.5° in the XRD pattern of all B-CMs, which was also confirmed by Raman spectroscopy analysis. While the D band was between 1340 and 1360 cm^–1^, the G band alternated between 1580 and 1600 cm^–1^ for all B-CMs. Moreover, the contact angle analysis results of the porous carbon obtained from twin-cocoon, fish scales, *Scaphium scaphigerum*, and fungi show that their structures have low contact angles. This is because nitrogen and oxygen groups found in these porous carbons significantly affect the hydrophilicity.

An active carbon with high surface area and mesoporous was synthesized from coconut shells by Uluganathan et al.^62^ In their research, after a ZnCl_2_ activation agent was added to coconut shells for 20 min at 275 °C, nitrogen gas was fed until 800 °C. Once the temperature reached 800 °C, CO_2_ gas also was transferred for 2 h. XRD analysis results show that the characteristic peaks of the carbon were approximately observed at 23 and 44°. The D and G peaks were 1349 and 1600 cm^–1^ in the Raman spectrum analysis, respectively. The results of these analyses indicated that the obtained active carbon had a large pore structure and a high graphitization value. In addition, it is seen from SEM analyses that the active carbon has a smooth surface morphology with a highly porous structure. Krikstolaityte et al.^63^ worked on activated carbon acquired from coffee beans. First, the pyrolysis process was applied to these coffee beans at 850 °C under a N_2_ atmosphere for 30 min, and the biochar was obtained as a nonactivated sample. Then, this product was activated in a steam–N_2_ atmosphere to investigate the effect of physical activation, and further research was conducted for three different periods such as 1, 2, and 3 h to investigate the effect of activation time. The final products obtained in three different processes were called AC1, AC2, and AC3, respectively. The researchers compared the obtained samples by performing XRD, SEM, and BET analyses. In the results of XRD analysis, two broad peaks were shown in all activated carbons (AC1, AC2, and AC3) at 23 and 43°, while a sharp peak was seen in BC at 27°. Two broad diffraction peaks displayed the presence of microcrystals, and a sharp peak revealed the presence of relatively large graphite crystallites. According to the SEM analysis, the presence of a well-developed porous structure was seen in AC3 compared to BC. This is because the adsorption and desorption of steam in the active site of the biochar causes the formation of hydrogen and oxygen-containing groups which increase the porosity and surface area of activated carbon.^83^ It is concluded that the steam activation and activation time contribute positively to the activated carbon morphology since more steam penetrates the biochar surface with the increase in the activation time. Liu et al.^64^ derived an active carbon from cuttlefish bones. The pyrolysis process was carried out under an argon atmosphere at 600 °C, and then the obtained carbon (MBPC) was exposed to air oxidation at 300 °C for 1, 3, and 8 h (MBPC-A1, MBPC-A3, MBPC-A8), separately. The peaks obtained from XRD results show that the peak values of MBPC-A1, MBPC-A3, and MBPC-A8 were closer to 22° than for MBPC. It was understood that defects in amorphous carbon resulting from air oxidation activation occurred and the graphitization degree of this carbon decreased. As a result of Raman analysis, D and G peaks of MBPC and MBPC-A3 products were 1350 and 1580 cm^–1^. The *I*_D_/*I*_G_ ratios were calculated as 0.94 and 1.00 for MBPC and MBPC-A3, respectively. This can be attributed to the fact that more oxygen entered the structure with the increase of oxygen groups after the activation process, and the defects in the structure increased with the decrease in carbon content. According to the SEM analysis of MBPC-A3, in which the best result was obtained, it was seen that the thickness of the carbon nanosheets and the surface roughness of the samples increased as the activation time increased.

Abbas et al.^65^ carried out pyrolysis at 600 °C for 3 h followed by chemical activation with a KOH activation agent at 800 °C for 1 h to derive activated carbon from black tea bags. Chemical reactions between KOH and carbon are given in eqs 2–6. The analyses were performed in detail by using various physicochemical measurement techniques for different impregnation ratios in order to evaluate the effect of the activation agent impregnation ratio on the activated carbons (AC2, AC3, and AC4: KOH/biochar mass ratios are 2, 3, and 4, respectively). It was seen from XRD results that a characteristic peak was exhibited at 43° as a shallow peak for all activated carbons and the most amorphous structure was observed in AC4 when compared to the surface structures of these carbons. These results indicated obviously that the surface structure of activated carbons improves by increasing the impregnation ratio. In Raman analysis, D and G bands for all carbons were exhibited as two broad bands at 1346 and 1597 cm^–1^, respectively. The *I*_D_/*I*_G_ ratios were 0.97, 1.03, and 1.01 for AC-2, AC-3, and AC-4, respectively. Furthermore, according to SEM analysis results, the highest porosity was seen in AC-4 due to the increment in macroporosity with the increase of impregnation ratio. Consequently, it is understood from all analysis results that the increase in the impregnation ratio of the activation agent favorably impacts the surface structure and porosity of the activated carbon due to the fact that the interaction between the activating agent and biochar increases.^79^
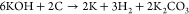
2
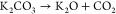
3
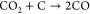
4
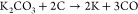
5
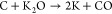
6

According to the study performed by Maharjan et al.^66^ a porous carbon having a high surface area was derived from orange peel by applying a chemical activation process under an argon atmosphere during 2 h at 800 °C with KOH activating agent. While the XRD results of the final product (OP-AC) obtained from this process showed that two separate broad peaks formed at around 29.5 and 43.0°, the D and G peaks observed from Raman analysis were approximately 1371 and 1604 cm^–1^, respectively. These results revealed that OP-AC, which was carbon in an amorphous and semigraphitic form, had a considerable quantity of defects and disordered structures. It was concluded from SEM analysis that the activated carbon had a well-developed porous structure. In another study, Maharjan et al.^67^ produced mesoporous carbon from Sal wood sawdust. After the HTC process was carried out at 275 °C with ZnCl_2_ activation agent for 20 min, a physicochemical activation process was applied to the hydrochar at 850 °C under a nitrogen atmosphere for 2 h. The morphological characteristics of the final product (SWD-AC) were thoroughly examined. According to the results of the XRD analysis, the characteristic peaks were found as two broad peaks at 30 and 42°. It is estimated from these peaks that SWD-AC is an amorphous semigraphitic carbon or a nanocrystalline carbon. From Raman analysis, the D and G peaks were observed at 1342 and 1577 cm^–1^, respectively, and the *I*_D_/*I*_G_ ratio was calculated as 0.99. These results showed that a material with a highly porous structure was obtained with the formation of irregular structures or defects in high-density graphite carbon. Moreover, it was seen from SEM analysis that SWD-AC was a porous material with a granular particle morphology at the nanoscale level. According to the aforementioned reviewed studies, it is understood that the improvement of surface morphology is highly dependent on the amount of activation agents, activation temperature, and activation time. Furthermore, it is important to determine the surface properties (surface area and pore volume) of the carbon materials as they have a significant impact on the performances of the VRFBs. In line with this result, a BET analysis of the reviewed studies is presented in Table 2. When the effect of activation agent used in synthesis stages is evaluated, it is seen from the table that the surface properties of the carbon materials activated with the KOH activation agent are higher than those activated with the other agents. The reason for this is that the gaseous products released from the chemical reactions given in eqs 2–6 improve the formation of a carbon porous structure, and so high-surface-area carbon materials can be obtained.

**Table 2:** BET Surface Analysis Results of Carbon Materials Synthesized from Various Biomass Sources

biomass	activation agent	inert gas	BET surface area (m^2^/g)	micropore surface area (m^2^/g)	mesopore surface area (m^2^/g)	total pore volume (cm^3^/g)	micropore volume (cm^3^/g)	mesopore volume (cm^3^/g)	ref
kiwifruit	FAC	Ar_2_	N/A	N/A	N/A	N/A	N/A	N/A	(51)
*Scaphium scaphigerum*	K_2_FeO_4_	Ar_2_	**1086.9**	N/A	N/A	N/A	N/A	N/A	(52)
persimmon	(NH_4_)_2_C_2_O_4_	Ar_2_	N/A	N/A	N/A	N/A	N/A	N/A	(53)
shaddock peel	H_3_PO_4_	Ar_2_	**882.7**	N/A	N/A	**1.99**	N/A	N/A	(54)
twin-cocoon	Na_2_CO_3_	Ar_2_	N/A	N/A	N/A	N/A	N/A	N/A	(55)
shrimp-shell-extracted chitin	N/Ar	N_2_	**616**	N/A	N/A	N/A	N/A	N/A	(56)
fungi	KOH	Ar_2_	**1412.7**	N/A	N/A	N/A	N/A	N/A	(57)
fish scales	KOH	Ar_2_	**1492**	**1386**	**106**	**0.84**	**0.69**	**0.15**	(58)
*Scaphium scaphigerum*	FeCl_3_	Ar_2_	**615.6**	**466.8**	**148.8**	**0.6**	**0.3**	**0.3**	(59)
*Scaphium scaphigerum*	urea	Ar_2_	**299.42**	N/A	N/A	N/A	N/A	N/A	(60)
sugar cane bagasse	KOH	N_2_	**1255 **	N/A	N/A	**0.537**	N/A	N/A	(61)
coconut shells		N_2_/CO_2_	**1652**	N/A	N/A	N/A	N/A	N/A	(62)
coffee beans		N_2_/steam	**1113**	N/A	N/A	**0.75**	**0.60**	**0.15**	(63)
cuttlefish bone		Ar_2_/air	**562.37**	N/A	N/A	N/A	N/A	N/A	(64)
black tea bags	KOH	N_2_	**2085**	**1930**	**155**	**1.174**	**0.97**	**0.204**	(65)
orange peel	KOH	Ar_2_	**1901**	**1407**	**494**	**0.94**	**0.59**	**0.35**	(66)
sal wood sawdust	ZnCI_2_	N_2_/CO_2_	**1857**	**577**	**1280**	**1.35**	**0.03**	**1.32**	(67)

When the surface areas of porous carbons derived from *Scaphium scaphigerum*(52,59,60) are analyzed, it is discovered that the activation agent has a considerable impact on their surface area. Namely, although the BET surface area is equal to 262.3 m^2^ g^–1^ in the case without agent, it is 1086.9 m^2^ g^–1^ with K_2_FeO_4_.^52^ While H_3_PO_4_,^54^ which is typically a dehydration agent, is used to increase porosity of a carbon material by removing oxygen and hydrogen from raw materials in water form, FeCl_3_^59^ is often used to increase the degree of graphitization of carbon materials. Also, the high surface area required to facilitate the mass transfer process can be obtained by the formation of nitrogen-doped carbon nanoparticles with urea,^60^ which is recognized as a nitrogen source. Although the same agent (KOH) is used for both biomasses of black tea and orange peel, a higher surface area of activated carbon from the tea bag resulted.

Another factor affecting the BET surface is the impregnation ratios of these agents. With increasing impregnation ratio, the BET surface area is reached by the development of pores and the enlargement of already-existing pores. It was reported in a study^53^ that the BET surface area rose from 7 to 16 m^2^ g^–1^ with the increase of impregnation rate of (NH_4_)_2_C_2_O_4_ from to 1 to 10. This increase can be seen also in another study.^65^ The BET surface area increased to 2085 m^2^ g^–1^ for a KOH impregnation ratio of 4. However, after this value, it began to decline, and the area was found to be 1556 m^2^ g^–1^ for an impregnation ratio of 5. The reason for this is that the increase of impregnation rate until an optimum value increases the surface properties. The use of more activating agents reduces the specific surface area and activation energy due to the higher chemical reaction leading to the destruction of the pores.^83^ Therefore, it can be concluded that the surface areas of carbon materials change with some parameters such as biomass precursor carbon content, carbonization and pyrolysis temperatures, and activation agent impregnation rate.

When BET surface analyses of active carbons obtained from physical activation^54−56^ are compared with each other, coconut shell has the highest surface area. The reason for this is thought to be the parameters affecting the activation process mentioned in Section 2, but the activation agent from these parameters has the most important effect on the physicochemical properties of the obtained carbon materials. In addition, according to the reviewed studies, BET surface areas for active carbons obtained from physical activation are lower than that from chemical activation because physical activation has a lower degree of carbon etching.^83^

**Figure 7:**
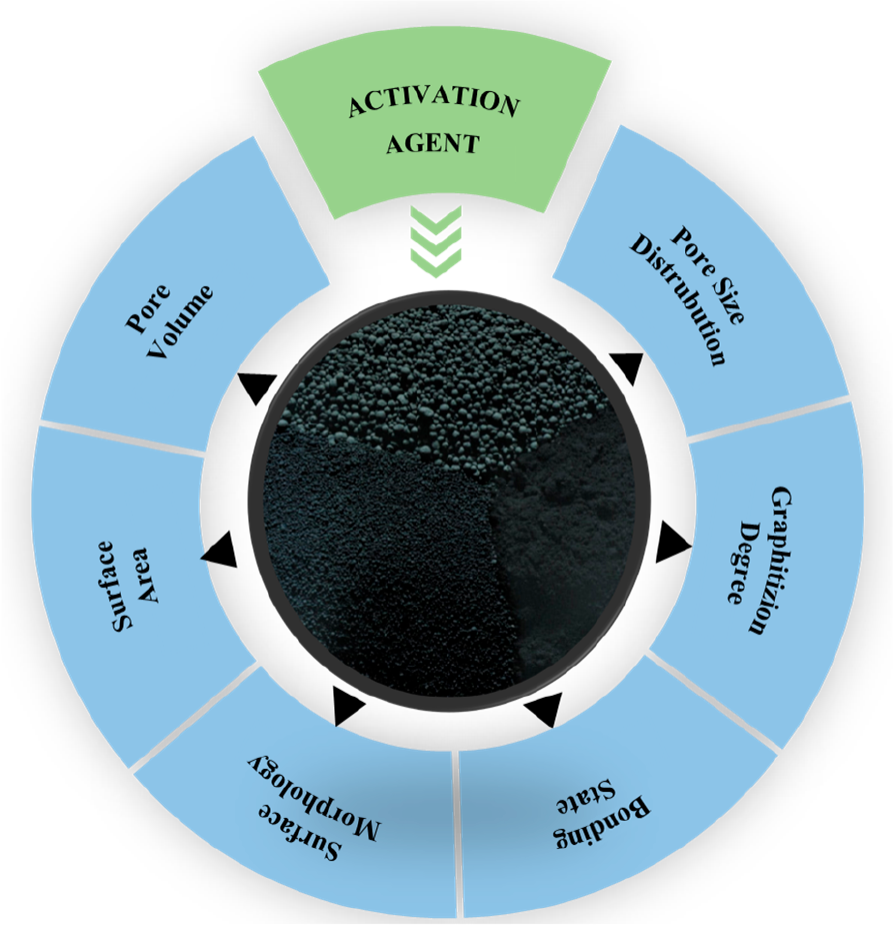
Physicochemical properties of the obtained carbon materials affected by the activation agent.

It is understood from the results of these studies that the activation agent significantly affects the pore size distribution, graphitization degree, and surface area of carbon materials as shown in Figure 7. Consequently, among the various activating agents, KOH is the most preferred in the literature due to lower activation temperature requirement, higher efficiency, and high surface area and pore volume improvement.^78,79^

## 5 Applications of Biomass-Derived Carbon Materials as an Electrocatalyst in VRFB

One of the key components affecting the performance and electrochemical behavior of a VRFB is the electrode, because the redox reactions occur on its surface. Therefore, the best electrode must have high catalytic activity, good conductivity, and high stability. The reaction kinetics and catalytically active sites of the most used carbon-based materials such as carbon felt, graphite felt, and carbon paper are low. In order to improve the electrochemical activity of these electrode materials, they are doped with carbon- or metal-based electrocatalysts through a variety of techniques, including doping catalysts and directly activating the surface of electrode materials. Furthermore, due to their positive properties, including environmental friendliness, low cost, good electrical conductivity, and renewable resources, B-CMs utilized in different energy storage applications have been extensively reported. The reported biomasses (such as kiwifruit, *Scaphium scaphigerum*, persimmon, etc.) used as electrocatalysts in VRFBs and their performance are reviewed in this study. The results of each study are evaluated individually here since the biomass precursors and activating agents play an important role in the potential performance of the porous carbons derived from biomass as also mentioned in the previous section.

**Figure 8:**
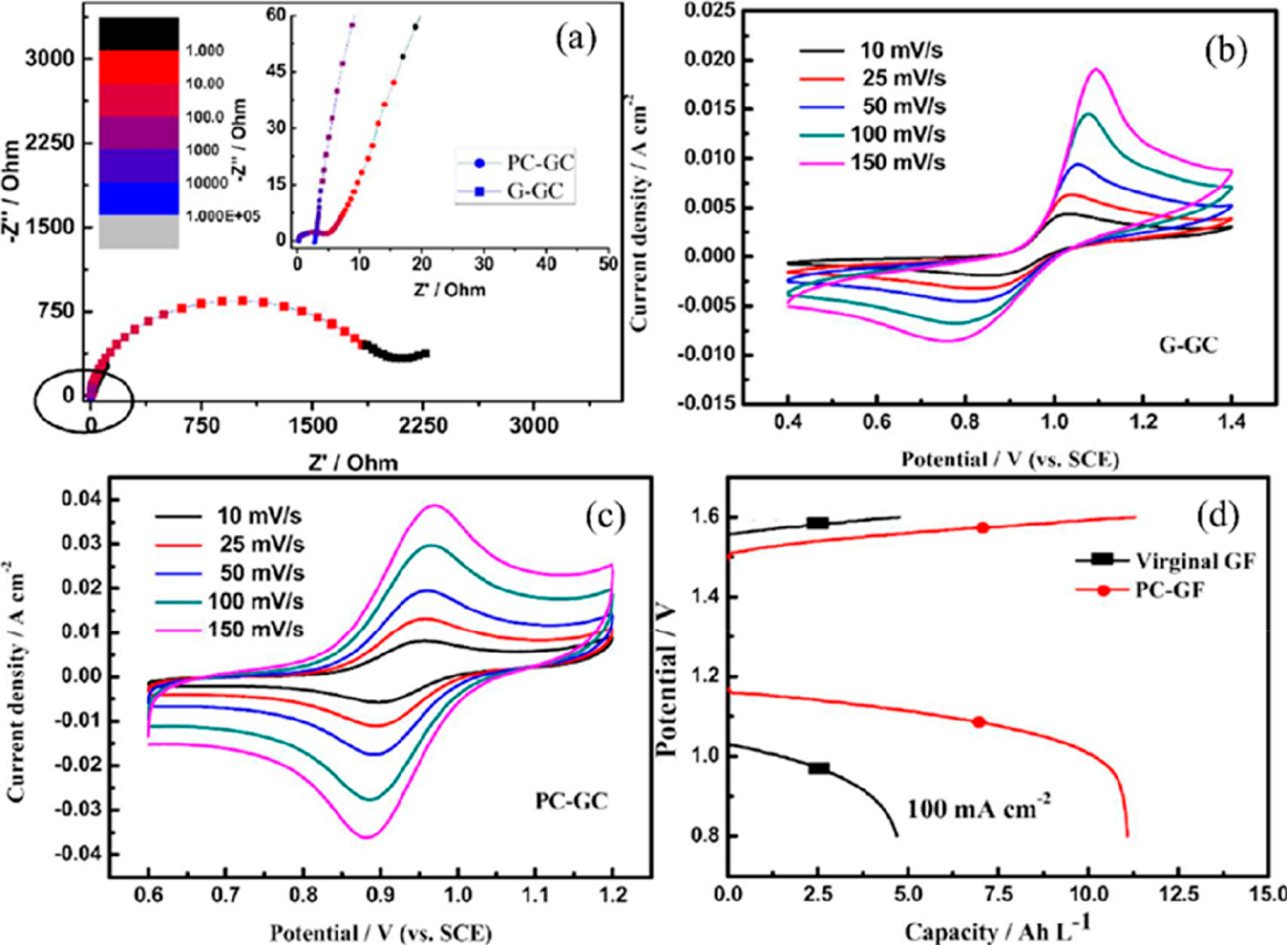
(a) EIS analysis, (b) CV results of the bare electrode at different scan rates, (c) CV results of the doped electrode at different scan rates, and (d) charge–discharge graphs for bare and modified electrodes at 100 mA cm^–2^. Reprinted with permission from ref (54). Copyright 2020, Elsevier,

Activated carbons, porous carbons, or N-doped carbons used to improve the performances of the batteries can be obtained using different biomasses as precursors in recent studies. Cheng et al.^51^ doped to a graphite felt electrode with N-doped porous carbon electrocatalyst derived from kiwifruit. They observed higher anodic and cathodic peak current densities in the battery cell using this electrocatalyst. The explanation for these high current densities is that the electrical conductivities of the electrodes increase with the addition of this catalyst. Furthermore, higher discharge voltage and a more stable discharge behavior were obtained from the battery constructed with N-doped porous carbon electrodes at different current densities (50–150 mA cm^–2^). When the porous carbon obtained from *Scaphium scaphigerum* electrocatalyst was doped to graphite felt by an immersion method,^52^ it was concluded that the reduction and oxidation peaks of the doped electrodes were improved by decreasing the overpotentials. Just as discharge capacity of the battery during 300 cycles decreases from 1599.6 to 923 mAh in the case of a doped electrode, it decreases from 1440 to 420 mAh in the case of a bare electrode. Moreover, the energy efficiency of the battery increased from 74% to 77.6% at 80 mA cm^–2^ due to enhanced reaction sites and charge transfer rates. In the case of using N-doped porous carbon produced from persimmon as an electrocatalyst in VRFBs,^53^ the electrode modified with the electrocatalyst had greater electrocatalytic performance and lower charge transfer resistance than the bare electrode in the redox reactions. As a result of this, the energy efficiency of the battery cell with graphite felt modified by the produced electrocatalyst is higher than that of the cell with the bare electrode. The porous carbon produced from shaddock peel was doped on a graphite felt electrode with an immersion method by Liu et al.^54^ As seen in Figure 8, the energy efficiency of a VRFB increased from 60% to 68.5% by decreasing activation, concentration, and ohmic overpotentials occurring in the battery electrodes with porous carbon. Wang and Li^55^ compared the effects of nitrogen- and oxygen-treated carbon materials derived from twin-cocoon on VRFB performance. Electrolyte accessibility, diffusion, and electrolyte utilization rate were increased with the increase of electrode hydrophilicity. As a result of the increase of functional groups with nitrogen and oxygen applications, it was observed that the activated sites increased and the energy efficiency of the battery increased from 60.7% to 72.5%.

**Figure 9:**
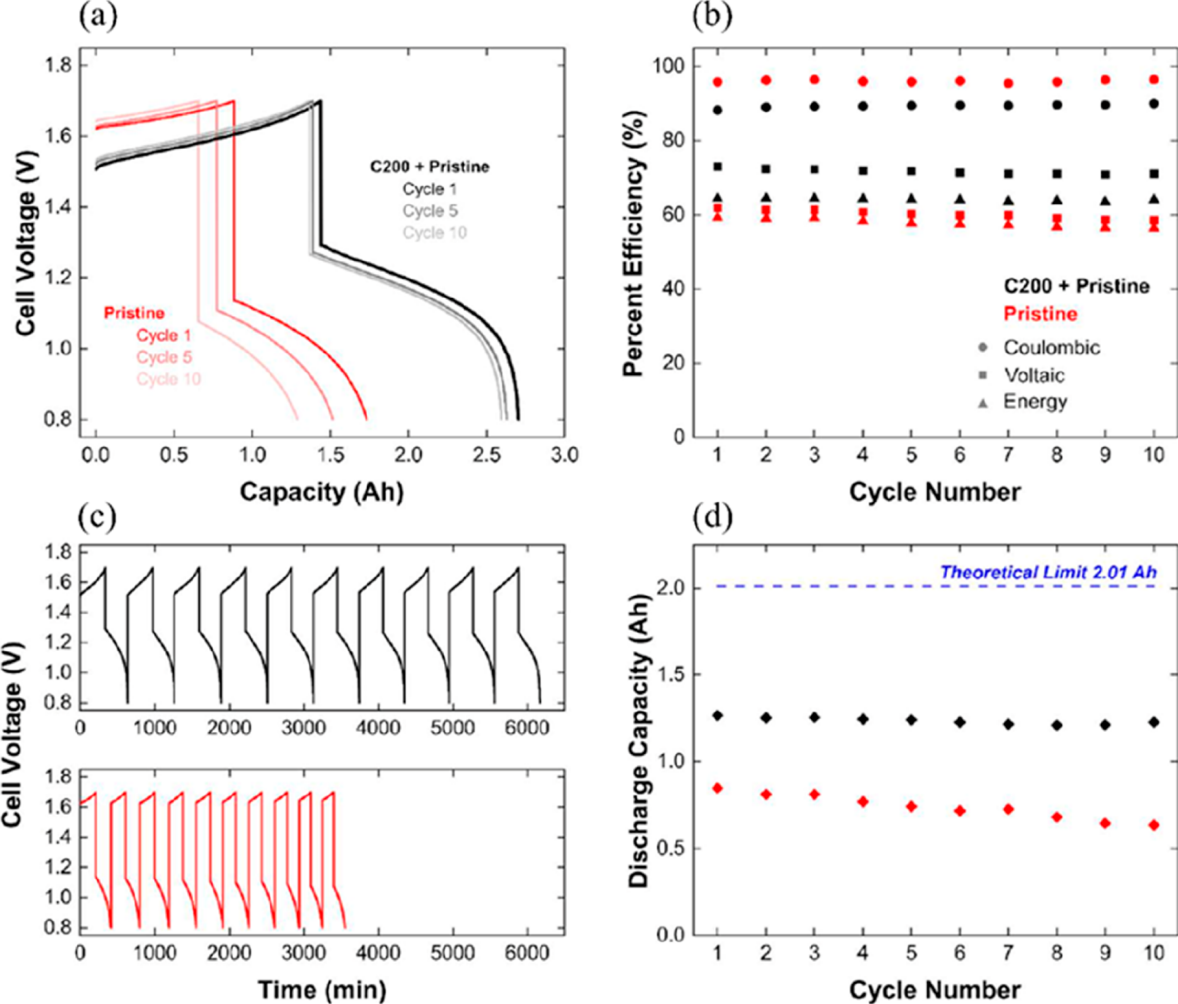
(a) Charge–discharge curves for the first, fifth, and 10th cycles. (b) Efficiency values during 10 cycles. (c) Continuous voltage profiles for pristine and modified electrodes. (d) Discharge capacities during 10 cycles All measurements were conducted at a current density of 100 mA cm^–2^. Reprinted with permission from ref (56). Copyright 2020, American Chemical Society,

Wan et al.^56^ doped active carbon derived from chitin to the graphite felt electrode. As seen in Figure 9a, the discharge voltage was reduced from 1.62 to 1.51 V by doping of a chitin-based electrocatalyst. The charge voltage decreased by around 6.5% with improved electrochemical behavior of the new electrode. As shown in Figure 9b,d, while the energy efficiency and discharge capacity of the battery equipped with a doped electrode was 64% and 1.22 Ah, respectively, these values were 56% and 0.64 Ah with the bare electrode. Also, the continuous voltage profiles of these cells are demonstrated in Figure 9c.

Jiang et al.^57^ obtained a porous carbon synthesized from fungi as electrocatalyst in a VRFB. Higher oxidation and reduction peak potentials were achieved from the electrode prepared by doping this electrocatalyst to a cloth electrode by the immersion method due to the electrode’s higher electrochemical activity. Moreover, the doped electrode had a higher and more stable discharge capacity and energy efficiency than the bare electrode due to the reduction of all overpotentials and electrocatalytic stability. With the result of the modification of graphite felt by doping with a carbon-based electrocatalyst produced from fish scale the ohmic, electrochemical, and mass transfer polarizations of the electrode decreased.^58^ This was because the activation process with KOH in the production of the electrocatalyst enhanced the electrochemical surface area of the carbon-based electrocatalyst. In addition, while the discharge capacity of the battery with a bare electrode was 89 mAh at the current density of 100 mA cm^–2^, its value for the doped electrode was 101 mAh.

A porous graphitic carbon acting as an electrocatalyst for vanadium redox reactions taking place at the interface between the electrode and electrolyte was obtained from *Scaphium scaphigerum* by a hydrothermal method and a subsequent Fe etching method.^59^ It was observed from the study that the mean discharge voltage of the electrode was higher than that of the bare electrode at all current densities (50–100 mA cm^–2^) because of the reduction of the electrochemical polarizations. The use of more graphitized electrocatalyst facilitates the charge and mass transport in the felt electrode. Moreover, while the energy efficiency of the battery equipped with a bare electrode was approximately 63.4%, the energy efficiency value increased to 69.9% at a current density of 100 mA cm^–2^ for the battery constructed with porous graphitic carbon electrodes. Furthermore, N-doped carbon electrocatalyst derived from *Scaphium scaphigerum* was also investigated by Jiang et al.^60^ The obtained electrocatalyst was used as a negative electrocatalyst in the battery cell. The results showed that the electrochemical polarization of the battery cell significantly decreased with utilization of N-doped carbon electrocatalyst. Therefore, the discharge capacity and energy efficiency of the battery cell increased at 150 mA cm^–2^. Mahanta et al.^61^ synthesized activated carbon from sugar cane bagasse and used it as an electrocatalyst in the positive electrode. The electrochemical surface area value of the improved electrode was increased 80 times compared with the thermally treated graphite felt. It is seen from the results that the improved electrode exhibited high electrocatalytic activity, and the energy efficiency of the battery cell constructed with this improved electrode was higher than that of the bare battery cell. A high-surface-area mesoporous carbon derived from coconut shell to improve the electrochemical characteristics of the electrode was used in electrodes as an electrocatalyst by Ulaganathan et al.^62^ It was revealed that the charge–discharge behavior of the improved electrode and the battery performance were enhanced with the increase of electrode reversibility. In a study^63^ carried out for the graphite felt electrode doped with activated carbon derived from spent coffee beans, it was found that the electrical conductivity of the electrode was improved with the addition of the developed electrocatalyst. In addition, the battery cell had a strong electrochemical performance as well as high energy and voltage efficiency when this electrocatalyst was used in the electrodes. When the porous carbon derived from cuttlefish bone was used as an electrocatalyst to improve the performance of the electrode,^64^ electrochemical analyses revealed that the modified electrodes had better catalytic activity than the bare electrode and the discharge capacity of the modified battery cell was considerably enhanced. Moreover, while the energy efficiency of the battery cell with the bare electrode was nearly 60%, that of the battery cell using modified electrodes increased to 70% at constant current density. For the battery consisting of the electrocatalyst modified by doping the activated carbon produced from waste black tea bags on graphite felt, the anodic and cathodic peak current densities increased because of the modified electrode’s high degree of microporosity and higher specific surface area.^65^ In addition, the modified electrodes demonstrated a stronger electrochemical performance than the bare graphite electrode. Maharjan et al.^66^ synthesized cost-effective activated carbon from orange peel and coated this activated carbon on a graphite bipolar plate. According to their results, the newly developed graphite bipolar plate demonstrated strong electrocatalytic activity due to the high surface area of activated carbon, which allowed for good contact between the electrode and the bipolar plate. At all current densities, the energy efficiency of the battery cell increased with a decrease in all overpotentials. When the same doping method was used for coating the highly mesoporous carbon derived from a different biomass (Sal wood sawdust) on a graphite plate,^67^ the peak current density values increased due to the increased electroactivity of the graphite plate. In addition, the overpotentials were reduced by lowering the charge transfer resistances in both the negative and positive half-cells. While the voltage and energy efficiency values of the battery cell with the bare plate were 85.2% and 84.3%, respectively, these values of the battery cell with the new plate were measured as 86.7% and 85.4%.

Table 3 shows the doping methods and various properties of electrodes modified with biomass-based electrocatalysts. From this table, carbon materials used as electrocatalysts derived from biomass are porous carbon, N-doped porous carbon, and activated carbon. In order to increase the electrochemical activity of the electrodes and decrease all polarizations, they are doped with an electrocatalyst. The immersion method, slurry coating, and masking method were selected to dope these electrocatalyst on the electrode. Immersion, which is one of the most easily accessible and low-cost methods, is usually preferred so that the suspended ink solution containing the electrocatalyst can penetrate all over the thick felt electrode structure. In terms of the discharge capacity fade, it is clearly seen from Table 3 that the discharge capacity fade of the battery cell with the improved electrodes is higher than that of the battery cell using a bare electrode.

**Table 3:** Electrochemical Analysis Results for VRFBs Modified with Biomass-Derived Carbon Materials as Electrocatalysts

biomass	electrode	electrocatalyst	doping method	treatment	charge/discharge potential	CV solution	current density (mA cm^–2^)	discharge capacity fade (%)	ref
kiwifruit	graphite felt	N-doped porous carbon	immersion	thermal	0.7–1.7 V	1.6 M V^3+^ + 3 M H_2_SO_4_	50	bare: 1 cycle, 110 mAh; 50 cycles, 73.5 mAh	(51)
						1.6 M VO^2+^ + 3 M H_2_SO_4_		doped: 1 cycle, 129 mAh; 50 cycles, 96.5 mAh	
*Scaphium scaphigerum*	graphite felt	porous carbon	immersion	K–Fe etching calcination	N/A	N/A	50	bare: 1 cycle, 1441 mAh; 300 cycles, 407.7 mAh	(52)
								doped: 1 cycle, 1605.4 mAh; 300 cycles, 913.3 mAh	
persimmon	graphite felt	N-doped porous carbon	immersion	thermal and nitrogen treatment	N/A	0.8 M V^3+^ + 0.8 M H_2_SO_4_	50	bare: 1 cycle, 85 mAh; 50 cycles, 74 mAh	(53)
						0.8 M VO^2+^ + 3.0 M H_2_SO_4_		doped: 1 cycle, 108.8 mAh; 50 cycles, 98.1 mAh	
shaddock peel	graphite felt	porous carbon	immersion	N/A	0.8–1.6 V	0.1 M VOSO_4_ + 3 M H_2_SO_4_	N/A	N/A	(54)
twin-cocoon	organic based	N/A	N/A	nitrogen–oxygen	0.9–1.7 V	1.0 M VOSO_4_ + 3.0 M H_2_SO_4_	100	N/A	(55)
shrimp-shell-extracted chitin	graphite felt	activated carbon	N/A	N/A	0.8–1.6 V	0.1 M V(IV) in 0.1 M H_2_SO_4_ 1 M V(II) in 2 M H_2_SO_4_	100	bare: 1 cycle, 852 mAh; 10 cycles, 640 mAh	(56)
								doped: 1 cycle, 1265 mAh; 10 cycles, 1220 mAh	
fungi	graphite felt	porous carbon	immersion	KOH	0.7–1.7 V	2 M VO^2+^ + 3 M H_2_SO_4_	50	bare: 1 cycle, 4057 mAh; 50 cycles,: 2556.9 mAh	(57)
								doped: 1 cycle, 4549.2 mAh; 50 cycles, 3564.9 mAh	
fish scale	graphite felt	porous carbon	immersion	lyophilization, KOH etching carbonization	0.7–1.7 V	1.6 M V^3+^ + 3.0 M H_2_SO_4_	75	bare: 1 cycle, 106 mAh; 50 cycles, 70 mAh	(58)
								doped: 1 cycle, 120 mAh; 50 cycles, 97.5 mAh	
*Scaphium scaphigerum*	graphite felt	porous carbon	immersion	Fe etching carbonization	N/A	1.6 M V^3+^ + 3.0 M H_2_SO_4_ 1.6 M VO^2+^ + 3.0 M H_2_SO_4_	N/A	N/A	(59)
*Scaphium scaphigerum*	graphite felt	N-doped porous carbon	immersion	temperature treatment with urea	0.7–1.7 V	1.6 M V^3+^ + 3 M H_2_SO_4_	100	bare: 1 cycle, 1605 mAh; 150 cycles, 880 mAh	(60)
						1.6 M VO^2+^ + 3 M H_2_SO_4_		doped: 1 cycle, 1992.4 mAh; 150 cycles, 1439 mAh	
sugar cane bagasse	graphite felt	activated carbon	immersion	thermal	N/A	0.1 M VO_2_^+^ in 2.5 M H_2_SO_4_	N/A	N/A	(61)
coconut shells	carbon paper	activated carbon	N/A	N/A	0.5–1.5 V	1.7 M V^3.5+^ in 4 M H_2_SO_4_	N/A	N/A	(62)
coffee beans	graphite felt	activated carbon	N/A	N/A	0.9–1.6 V	1.6 M V^3+/4+^ (1:1 mixture of V^3+^ and V^4+^) + 4 M H_2_SO_4_	N/A	N/A	(63)
cuttlefish bone	graphite felt	porous carbon	N/A	thermal	0.7–1.65 V	1.6 M V^3+^ + 3 M H_2_SO_4_	50	bare: 1 cycle, 106.87 mAh; 50 cycles, 80.877 mAh	(64)
						1.6 M VO^2+^ + 3 M H_2_SO_4_		doped: 1 cycle, 117 mAh; 50 cycles, 98.73 mAh	
black tea bags	graphite felt	activated carbon	masking method	thermal	1.1–1.6 V	1.6 M V^3.5+^ in total 4.5 M sulfate solution	10	bare: 1 cycle, 16.2 mAh; 50 cycles, 2.84 mAh	(65)
								doped: 1 cycle, 30.3 mAh; 50 cycles, 13.2 mAh	
orange peel	graphite plate	activated carbon	slurry coating	N/A	0.9–1.6 V	1.6 M V^3.5+^ in 4.5 M total sulfate	N/A	N/A	(66)
Sal wood sawdust	graphite plate	activated carbon	slurry coating	N/A	0.9–1.65 V	1.6 M V^+3.5^ (4.5 M H_2_SO_4_)	N/A	N/A	(67)

**Figure 10:**
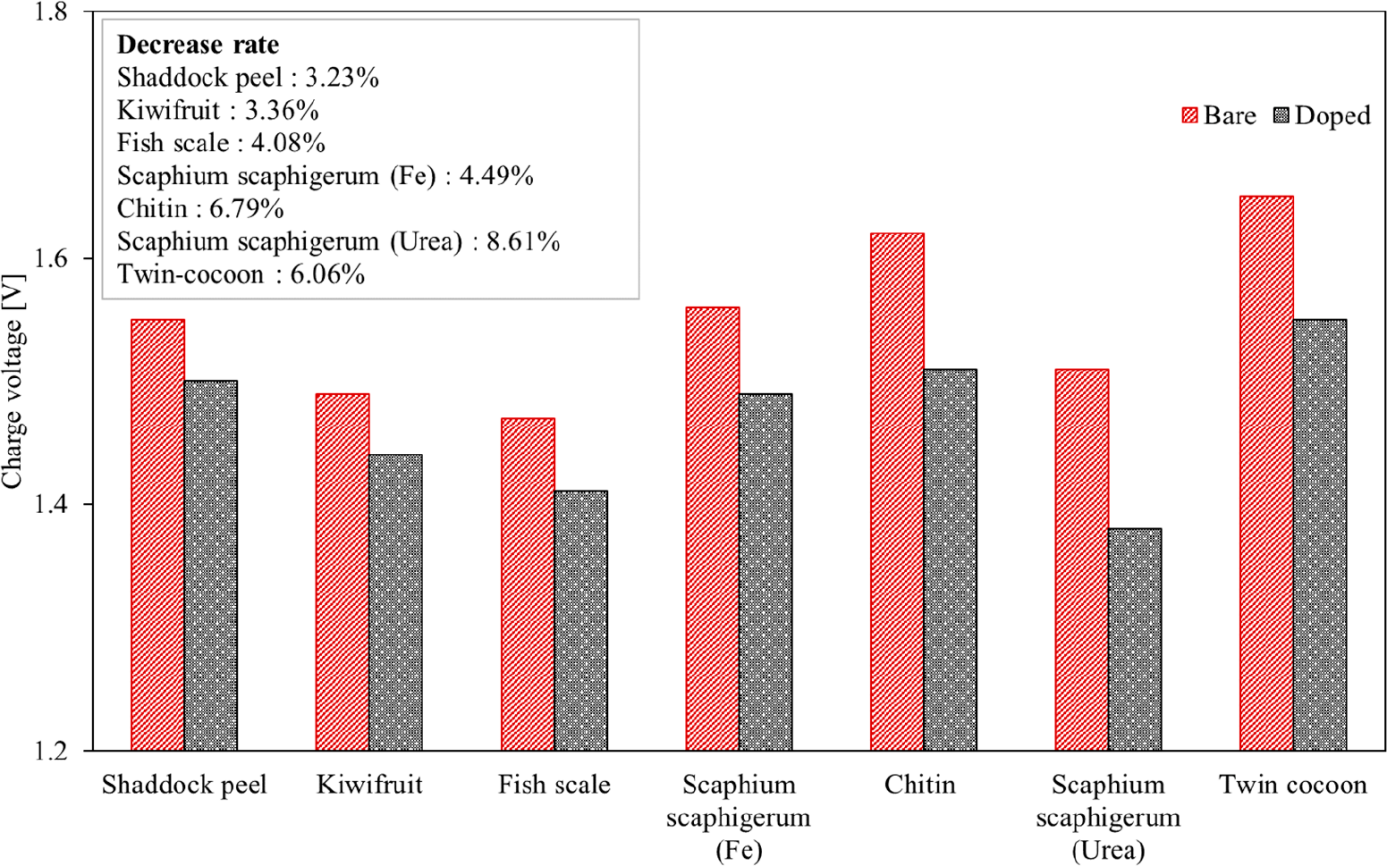
Variations of initial charge voltages with biomass-derived carbon electrocatalysts at a current density of 100 mA cm^–2^.

**Figure 11:**
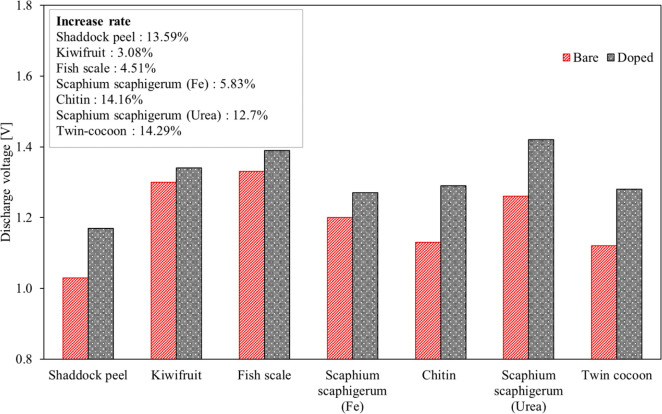
Variation of initial discharge voltage with biomass-derived carbon electrocatalysts at 100 mA cm^–2^.

The variations of initial charge and discharge voltages with use of biomass-derived carbon electrocatalysts at 100 mA cm^–2^ are shown in Figures 10 and 11, respectively. Each figure also shows the percent change value between the bare and doped electrodes. Due to different chemical and physical properties of electrodes, these properties cause different charge, ohmic, and mass transfer resistance. Therefore, the effect of the electrocatalyst modification on the charge–discharge voltage was compared according to the rate of the changes as well. It is known that the energy amount required for the charge process of the battery decreases with decreasing charge voltage value while the energy produced by the battery during the discharge process increases with increasing discharge voltage. In this respect, when charge and discharge voltages of each considered study are examined, the lowest charge and the highest discharge voltages are found for N-doped carbon materials obtained by using *Scaphium scaphigerum* and urea due to the superior effects of the electrocatalyst on the overpotentials. Therefore, it can be said that the effective precursor is *Scaphium scaphigerum* treated with urea, in view of the battery performance. For this doped case, the charge and discharge voltages are 1.38 and 1.42 V, respectively. In addition, while the charge voltages with doped electrodes are lower than those with bare electrodes, the discharge voltages are higher. As the maximum decrease rate in charge voltage can be seen in *Scaphium scaphigerum* (urea) of 8.61%, the maximum increase rate in discharge voltage is in the twin-cocoon of 14.29%.

**Figure 12:**
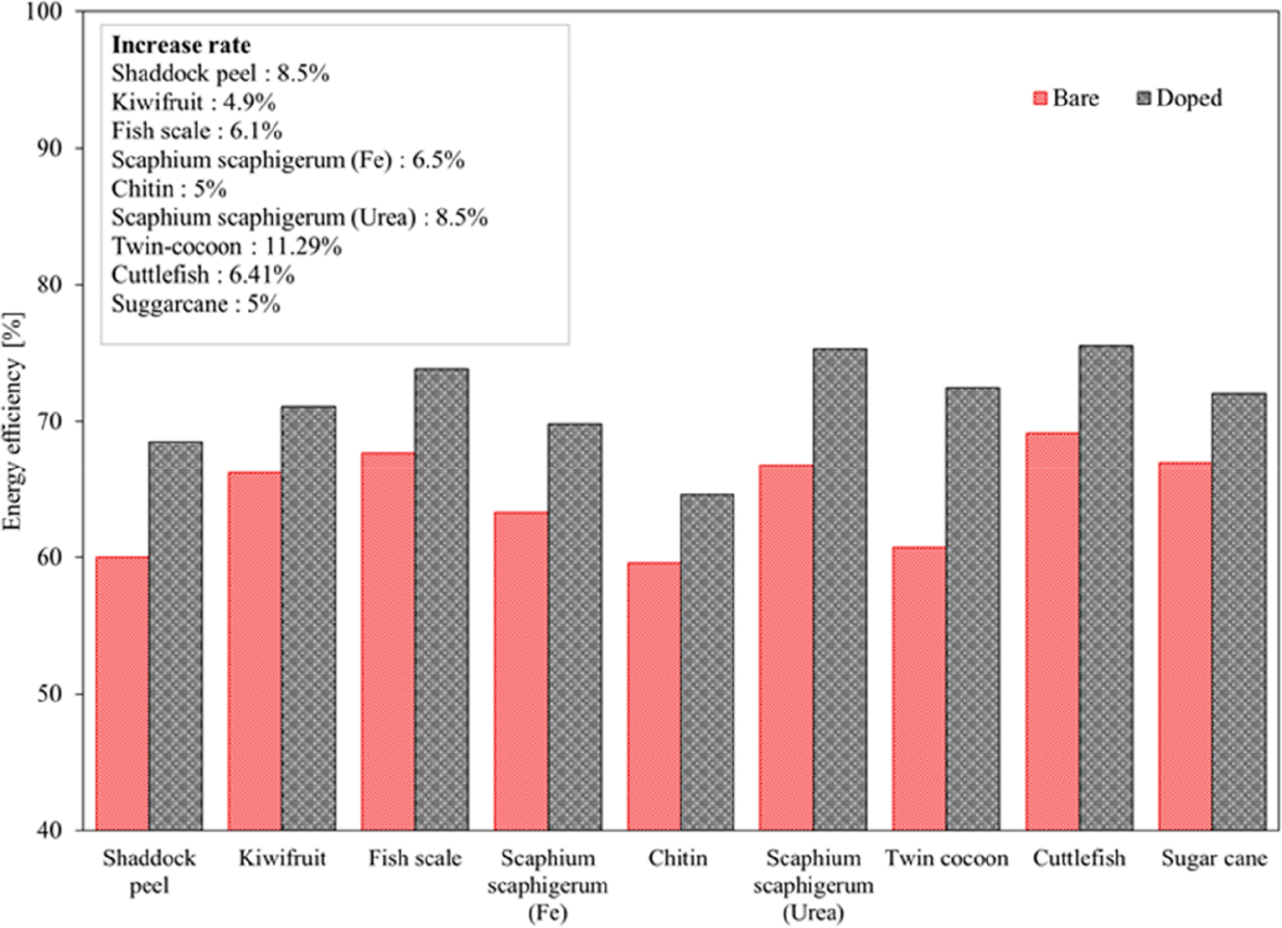
Variation of energy efficiencies with biomass-derived carbon electrocatalysts at 100 mA cm^–2^.

Another important parameter in batteries is energy efficiency, as well as battery performance. It shows the rate between the net energy change of the battery and energy input from the outside or output from the outside. The physical properties of the electrocatalyst, such as graphitization value, high porosity value, and electrochemical properties, directly affect charge and mass transfer polarization. Aside from charge–discharge voltages, the physical and electrochemical characteristics of the felt electrode and electrocatalyst have a significant impact on the value and the increased rate of energy efficiency. The variations of energy efficiencies of biomass-derived carbon electrocatalysts at 100 mA cm^–2^ are evaluated in Figure 12. The highest energy efficiency of 75% is observed in cuttlefish and *Scaphium scaphigerum* based carbon electrocatalysts. When examined in terms of the energy efficiency increase rate of the doped electrocatalyst over the bare electrocatalyst, the highest rate is obtained as 11.29% in twin-cocoon based electrodes. The energy efficiencies were increased in all studies because the electrocatalyst modifications decreased the overpotentials in the electrode and improved the electrocatalytic activation.

## 6 Conclusions

Although the superior properties of VRFBs such as high energy efficiency, scalability, eco-friendliness, and safety have drawn attention as a large electrochemical energy storage system, they have low power density. In order to improve this, many researchers have been trying to increase the electroactivity of electrodes with different applications and electrocatalyst additives. Among all the studied electrocatalysts up to now, biomass-derived carbon based electrocatalysts are seen as promising candidates because of their many advantages such as renewability, abundance in nature, sustainability, and chemical structure properties. Within the scope of this paper, biomass-derived porous carbon electrocatalysts researched in the literature were compiled, and their synthesis methods and performance as a result of using them as electrocatalysts in VRFBs were presented in detail. The main results in the reviewed studies can be listed as follows.

1. Among the porous carbons synthesized from the biomass source, the most promising electrocatalyst is one derived from black tea bags due to the fact that this electrocatalyst has a high surface area and porous structure according to the BET analysis result. The reason for this is due to the KOH activating agent, which provides the formation of oxygen-containing groups and the porous structure.
2. The lowest initial charge and the highest initial discharge voltages were obtained for the electrode doped with the carbon derived from *Scaphium scaphigerum* as 1.38 and 1.42 V, respectively.
3. On comparison of the rate of change in voltages of the bare and doped electrodes, the highest discharge voltage increase rate is equal to 14.29% in the case of twin-cocoon and the highest charge voltage decrease rate is 8.61% in the case of urea-treated *Scaphium scaphigerum*.
4. The maximum energy efficiency was achieved in a VRFB equipped with electrodes doped with the carbon derived from *Scaphium scaphigerum* and cuttlefish, and its value is 75% at a constant current density of 100 mA cm^–2^. However, the highest increase rate of 11.29% was observed for the battery with the electrode doped with carbon derived from twin-cocoon.
5. It has been observed that the electrochemical performances of electrocatalyst-doped electrodes are improved in all studies compared to bare electrodes. However, it can be said also that the level of this improvement for the same electrodes is directly dependent on the electrochemical properties of the electrocatalyst.

Consequently, considering the positive results of the reviewed studies, it is obvious that the use of environmentally friendly biomass-derived carbon materials as electrocatalysts in VRFBs significantly contributes to the sustainability and the cost effectiveness of the batteries.

## Abbreviations

VRFB: vanadium redox flow battery
RFBs: redox flow batteries
B-CMs: biomass-derived carbon materials
HTC: hydrothermal carbonization
XRD: X-ray diffraction
BET: Brunauer–Emmett–Teller
CV: cyclic voltammetry
EIS: electrochemical impedance spectroscopy
FAC: ferric ammonium citrate
